# Interface dermatitis in a patient with TRNT1 deficiency: A case report

**DOI:** 10.1016/j.jdcr.2025.02.039

**Published:** 2025-03-20

**Authors:** Andrew J. Gauger, Danielle Y. Del Mundo, Anita N. Haggstrom, Ahmed K. Alomari

**Affiliations:** a Department of Dermatology, Indiana University School of Medicine, Indianapolis, Indiana; b Department of Pathology, Indiana University School of Medicine, Indianapolis, Indiana

**Keywords:** autoinflammatory, case report, congenital sideroblastic anemia, cutaneous eruption, immunodeficiency, interface dermatitis, SIFD, TRNT1 deficiency

## Introduction

A syndrome of sideroblastic anemia with B-cell immunodeficiency, periodic fevers, and developmental delay was first described in 2013^1^ and later was found to be driven by mutations in TRNT1.^2^ This gene encodes transfer RNA nucleotidyl transferase 1, an enzyme critical for protein biosynthesis. In subsequent years, several studies have elucidated the vast heterogeneity in both the observed TRNT1 gene mutations and their respective clinical phenotypes.^3^, ^4^, ^5^ Additionally, work has been done investigating the auto-inflammatory phenotype^6^^,^^7^ seen in this disease entity. Most patients experience recurrent inflammatory episodes, developmental delay, and sideroblastic anemia with B-cell deficiency with hypogammaglobulinemia.^3^ Affected children generally exhibit multiorgan involvement, including gastrointestinal, ocular, and muscular manifestations. However, cutaneous manifestations are less commonly described^3^ but include aseptic panniculitis,^7^ erythema nodosum,^4^ desquamating plaques,^8^ and neutrophilic dermatoses.^9^ Previously, a case of erythema nodosum in a patient with TRNT1 deficiency was associated with an interface dermatitis pattern on histopathologic analysis.^4^ Herein, we report a case of TRNT1 mutation resulting in a cutaneous eruption characterized by an interface dermatitis on histology.

## Case presentation

**Fig 1:**
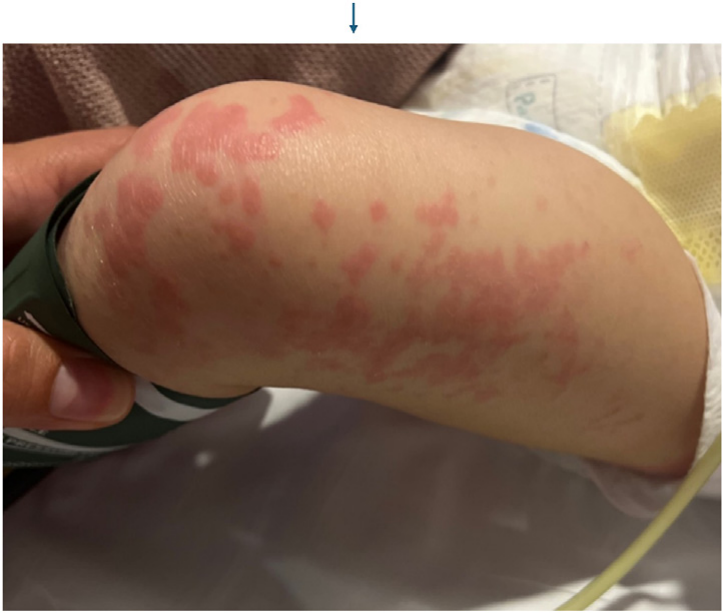
Clustered *dark pink* papules and plaques on the left lower extremity on day of consultation.

**Fig 2:**
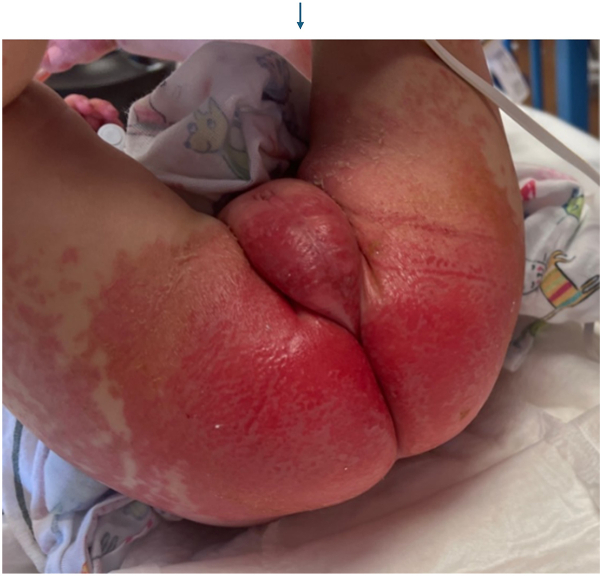
Confluent *bright red* plaques on buttocks and scrotum with patchy *red* plaques on thighs 3 days after consultation.

**Fig 3:**
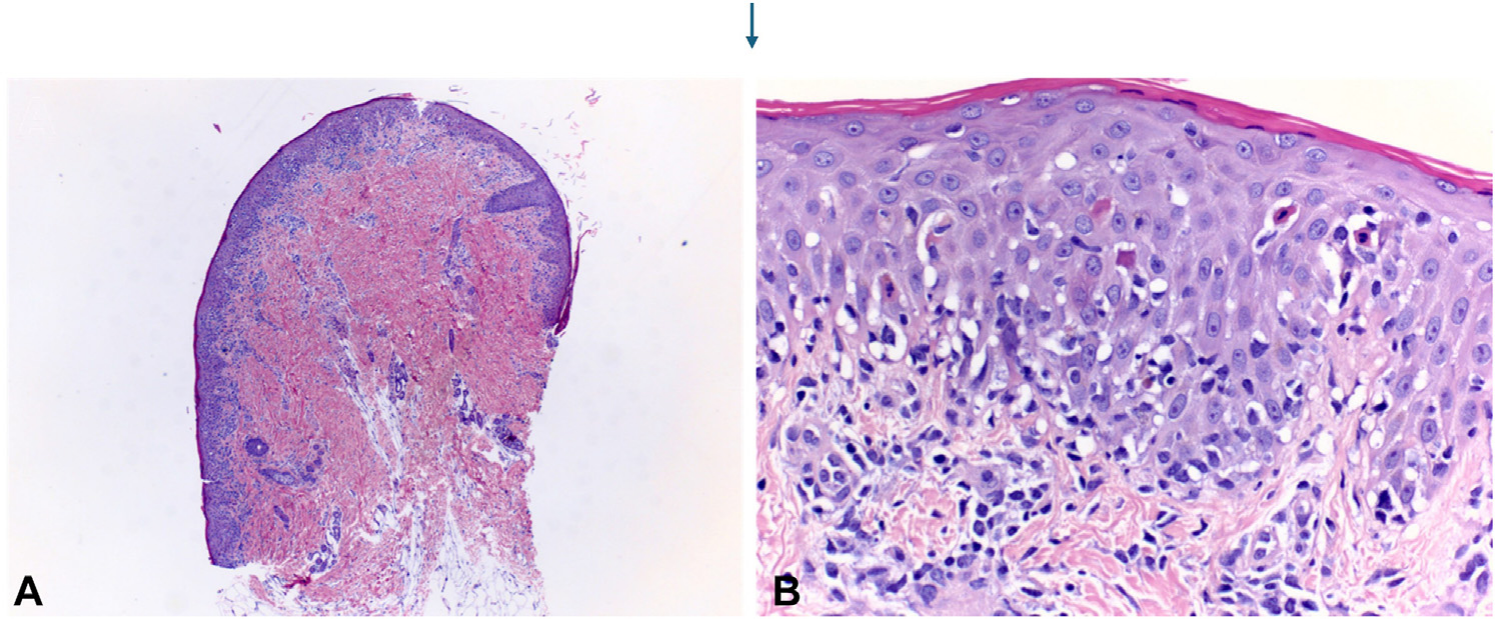
**A,** (40×) and (**B**) (400×), with (**A**) showing a superficial delicate band-like lymphocytic infiltrate while (**B**) shows vacuolar interface damage with scattered dyskeratotic cells and rare satellite cell necrosis.

**Fig 4:**
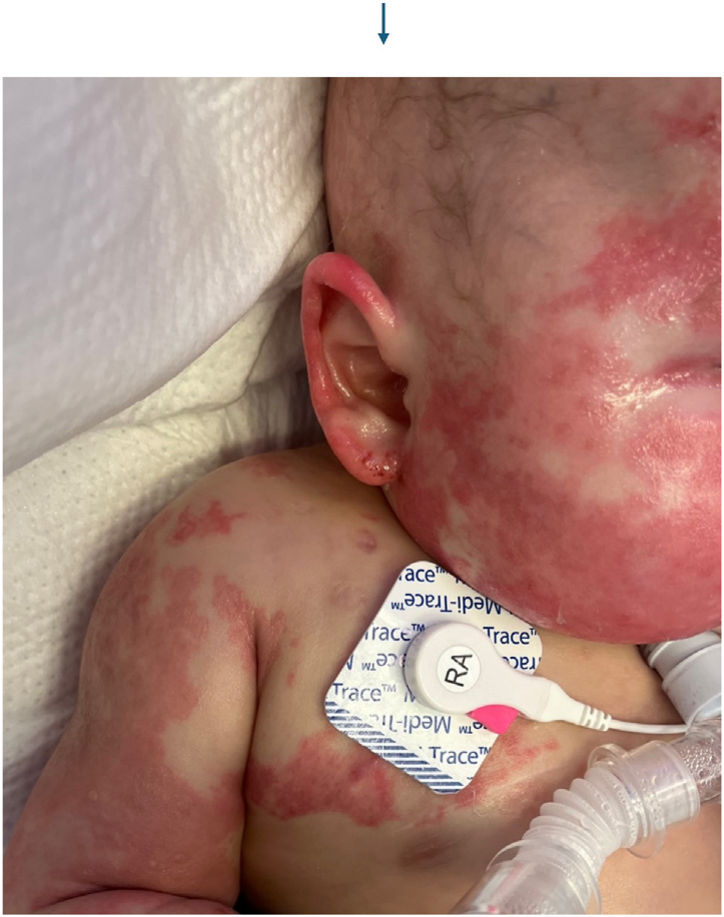
Patchy *pink* confluent plaques on the face, ear, chest, and arm 3 days after etanercept initiation.

An 18-month-old boy with a history of B-cell immunodeficiency secondary to TRNT1-related mitochondrial disorder (c. 668T>C) on intravenous immunoglobulin (IVIG) therapy presented with a new cutaneous eruption. He had a 1-year history of recurrent hypoalbuminemia coinciding with inflammatory episodes, for which rheumatology was managing with intermittent etanercept. Three weeks prior to admission, he presented with itchy plaques affecting his trunk and arms which had been managed by his allergist and had progressed despite treatment with triamcinolone 0.1% ointment, nystatin cream, oral hydroxyzine, and a 5-day course of oral fluconazole 10 mg/kg. The eruption spread to the neck and head with increased scale over the next 2 weeks. He was subsequently admitted to the hospital for hypoalbuminemia and worsening eruption. Initial physical exam revealed large coalescing scaly plaques involving the forehead, inguinal folds, and scrotum, along with few well-defined, smooth red papules on the left leg (Fig 1). Differential diagnosis included psoriasis, seborrheic dermatitis, cutaneous candidiasis, CARD14 associated papulosquamous eruption, neutrophilic dermatoses, and acrodermatitis enteropathica. The plaques worsened over the next 3 days with confluent spread to the bilateral buttocks (Fig 2), scrotum, and posterior scalp despite twice daily application of ketoconazole 2% cream and triamcinolone 0.1% ointment. Fungal and bacterial cultures were negative, and zinc levels were normal at 111.4. A punch biopsy from a left leg plaque revealed a lymphocytic interface dermatitis with scattered dyskeratotic cells, basal vacuoles, and focal hyper-parakeratosis (Fig 3). An autoinflammatory immune mediated etiology was favored in the context of the patient’s TRNT1 deficiency. Negative lupus serologies argued against other causes of interface dermatitis such as Rowell syndrome and drug-induced cutaneous lupus. Etanercept 7.5 mg weekly was initiated by rheumatology for both his concurrent autoimmunity symptoms and interface dermatitis (Fig 4). Initial clearing of the eruption began on the posterior scalp and inguinal area, and by day 7 on etanercept, the plaques had resolved with post-inflammatory hyperpigmentation. However, the patient then developed intestinal pneumatosis requiring systematic antibiotic therapy and etanercept was paused. One month later, the eruption recurred in a similar distribution when the patient was re-admitted for sepsis. Repeat laboratory and histopathologic analysis was consistent with prior hospital stay, and the eruption cleared with topical steroids despite being off etanercept during this flare.

## Discussion

Skin findings in patients with TRNT1 mutations, including aseptic panniculitis,^7^ erythema nodosum,^4^ desquamating plaques,^8^ and neutrophilic dermatoses^9^ have been reported. All of these patients first had a history of recurrent febrile and inflammatory episodes arising shortly after birth.

One case details an aseptic, erythematous nodule of the limb arising during a febrile episode.^8^ Histopathologic analysis revealed lobular and septal neutrophilic panniculitis. No recurrence of cutaneous symptoms were seen after several years of IVIG treatment. Another report presented 2 patients with TRNT1 deficiency,^9^ one developed painful, edematous desquamating plaques of the hands, while the other patient developed painless subcutaneous nodules on the extensor lower extremities. Both patients failed systemic steroids and had resolution of cutaneous symptoms and inflammatory episodes on etanercept. A third case described a painless eruption of firm pink papules and nodules affecting the trunk and limbs. Histopathology demonstrated an intense dermal neutrophilic infiltrate, and cutaneous clearance was observed with subcutaneous immunoglobulin and etanercept treatment.^9^ One final case reported a erythema nodosum-like eruption occurring during a febrile episode.^4^ Histopathologic analysis revealed septal panniculitis consistent with erythema nodosum in addition to interface dermatitis with vacuolar changes to the basal layer and dyskeratotic cells. IVIG was later initiated for hypogammaglobulinemia but did not improve the cutaneous symptoms, however initiation of etanercept treatment alongside IVIG later relieved the febrile episodes and cutaneous findings.

Interestingly, mutations in TRNT1 manifest with both immunodeficiency and autoinflammatory sequelae. Notably, sideroblastic anemia with B cell immunodeficiency, fevers, and developmental delay syndrome resulting from TRNT1 mutation is considered a systemic autoinflammatory disorder. Here, inborn errors of immunity cause an imbalance in the innate immune system, thereby driving an inflammatory cascade.^10^ TRNT1 is believed to play a critical role in protein homeostasis; in similar disorders like CANDLE syndrome, abnormal protein homeostasis leads to accumulation of proteins and lysis of cells thereby driving inflammation.^7^ FARSA deficiency, which encodes an aminoacyl tRNA synthetase, has also been associated with hypoalbuminemia and has a similar pattern of elevated inflammatory markers as seen in TRNT1 deficiency.^10^ Upregulated neutrophil-related genes, increased reactive oxygen species in fibroblasts, and increased cytokines including interleukin-6 and interferon gamma have all been reported in TRNT1 deficiency.^6^^,^^7^ An interface dermatitis pattern on histopathology has also been documented in Aicardi-Goutieres syndrome, another autoinflammatory disease.^11^ We present our case as the second reported interface dermatitis in a patient with TRNT1 deficiency with a different clinical morphology. Like other reports, improvement in cutaneous symptoms was seen with initiation of etanercept treatment along with existing IVIG therapy and topical corticosteroids, supporting a possible autoinflammatory etiology in this disease entity.

## Conflicts of interest

None disclosed.
